# Supplementary material for: The Effect of the Periodic Drying Method on the Drying Time of Hazelnuts and Energy Utilization
Source: Foods. 2024 Mar 15;13(6):901. doi: 10.3390/foods13060901 (PMC10969507; doi:10.3390/foods13060901)
Supplement: Supplementary file 1 [file foods-13-00901-s001.zip › foods-2895384-supplementary.pdf]

The remaining results for case 2, 3, 4, 5, 7, 8, 9, 10, 12, 13, 14 15 are presented as a supplementary file.

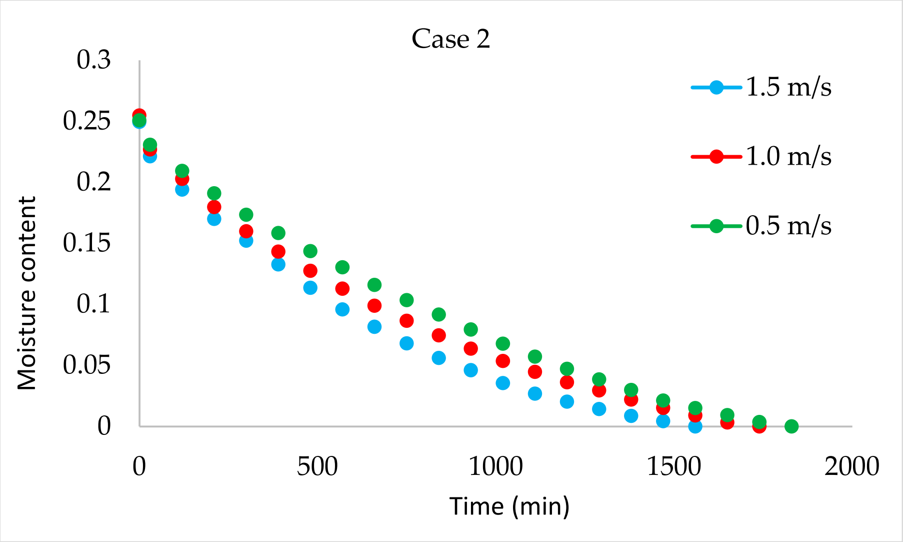

Figure S1. Change of time-dependent moisture content with respect to air velocities during case 2.

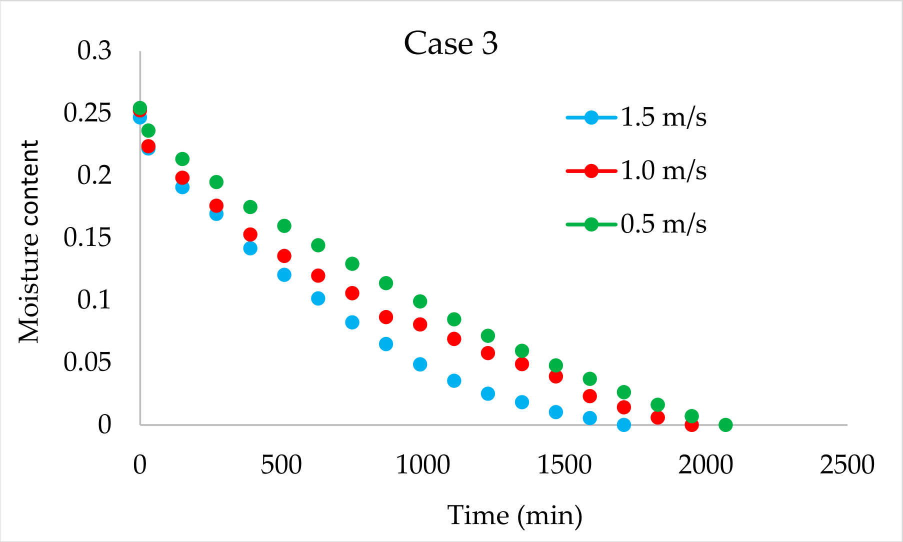

Figure S2. Change of time-dependent moisture content with respect to air velocities during case 3.

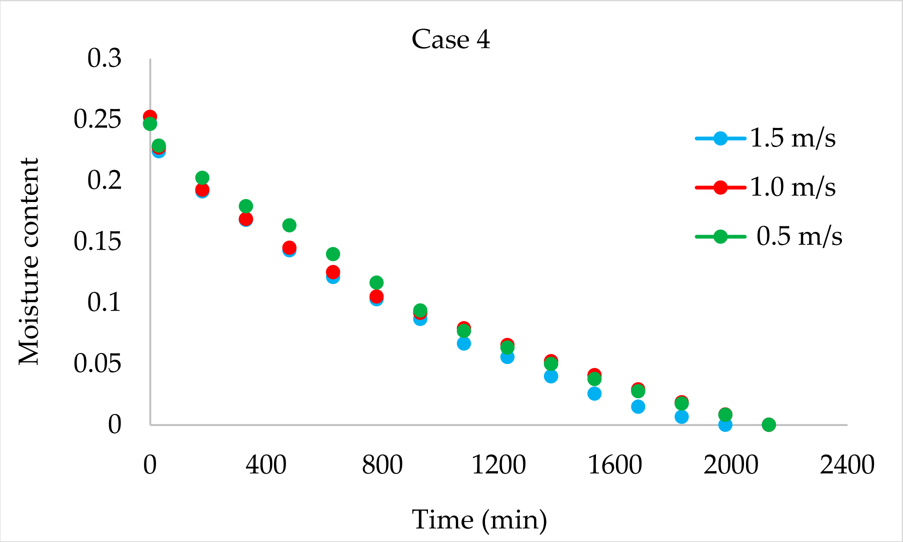

Figure S3. Change of time-dependent moisture content with respect to air velocities during case 4.

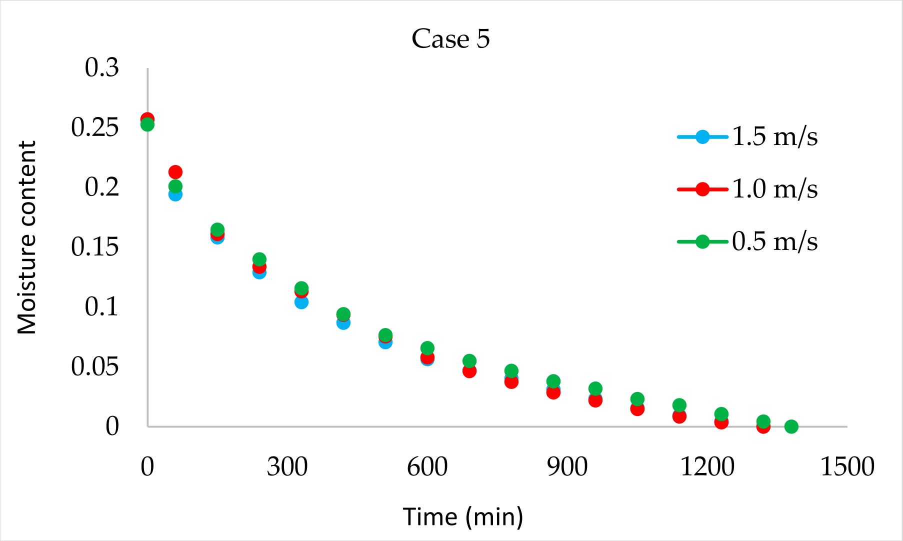

Figure S4. Change of time-dependent moisture content with respect to air velocities during case 5.

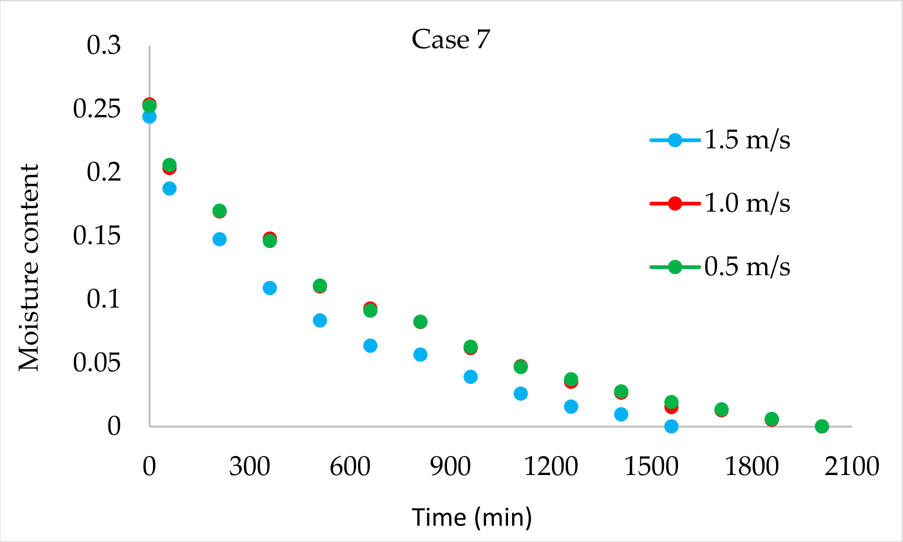

Figure S5. Change of time-dependent moisture content with respect to air velocities during case 7.

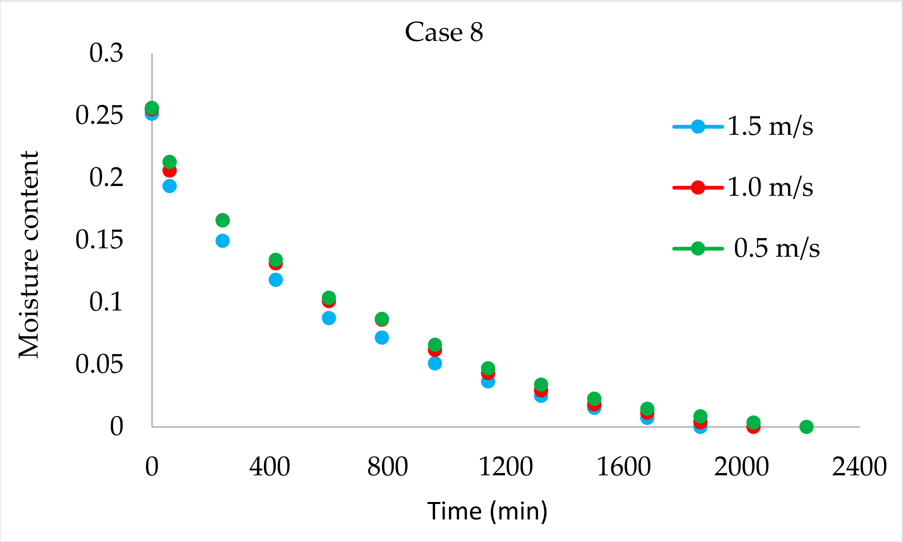

Figure S6. Change of time-dependent moisture content with respect to air velocities during case 8.

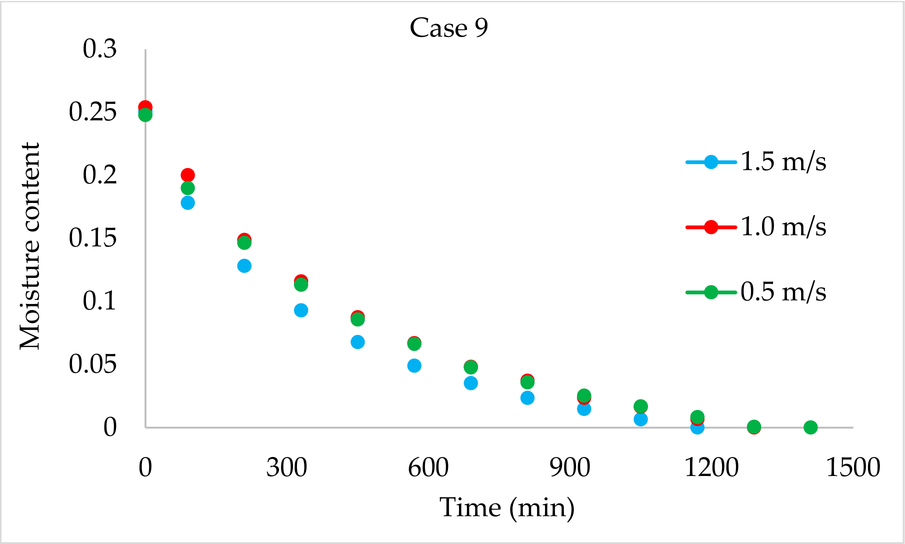

Figure S7. Change of time-dependent moisture content with respect to air velocities during case 9.

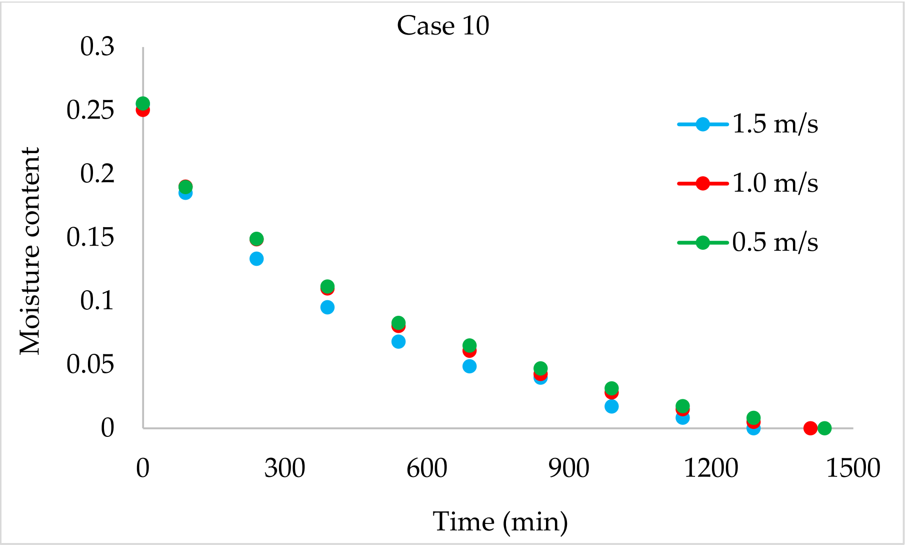

Figure S8. Change of time-dependent moisture content with respect to air velocities during case 10.

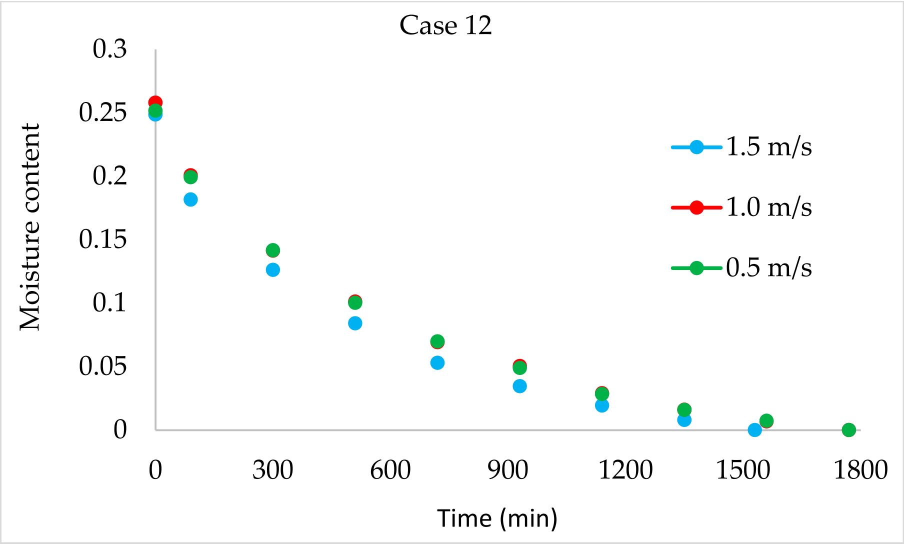

Figure S9a. Change of time-dependent moisture content with respect to air velocities during case 12.

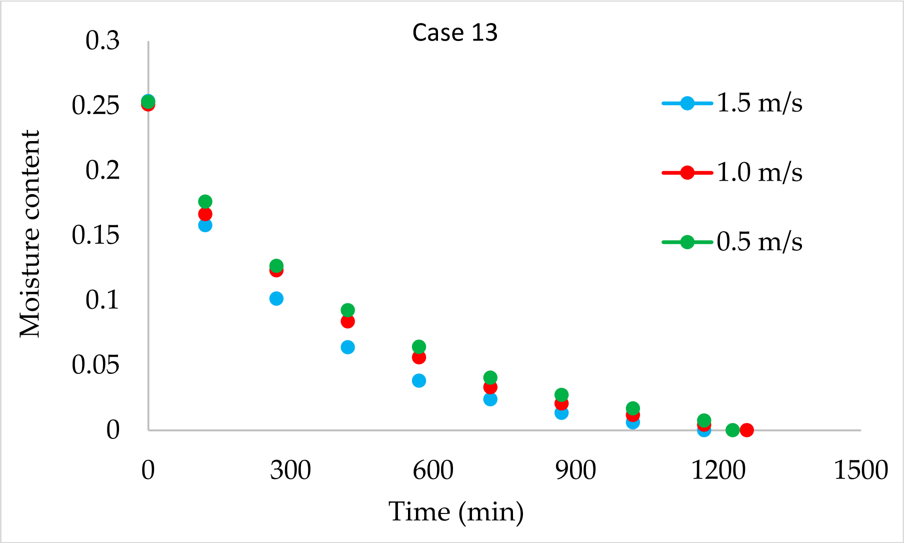

Figure S9b. Change of time-dependent moisture content with respect to air velocities during case 13.

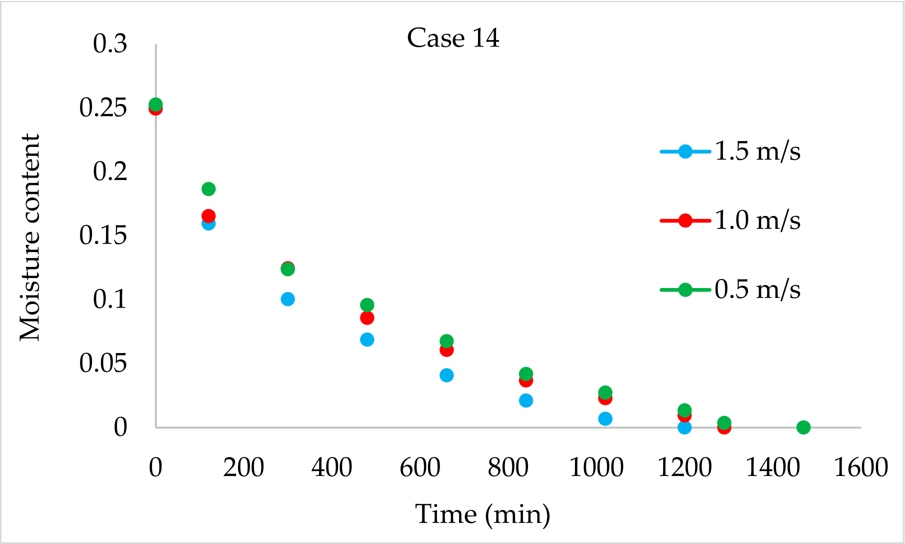

Figure S10. Change of time-dependent moisture content with respect to air velocities during case 14.

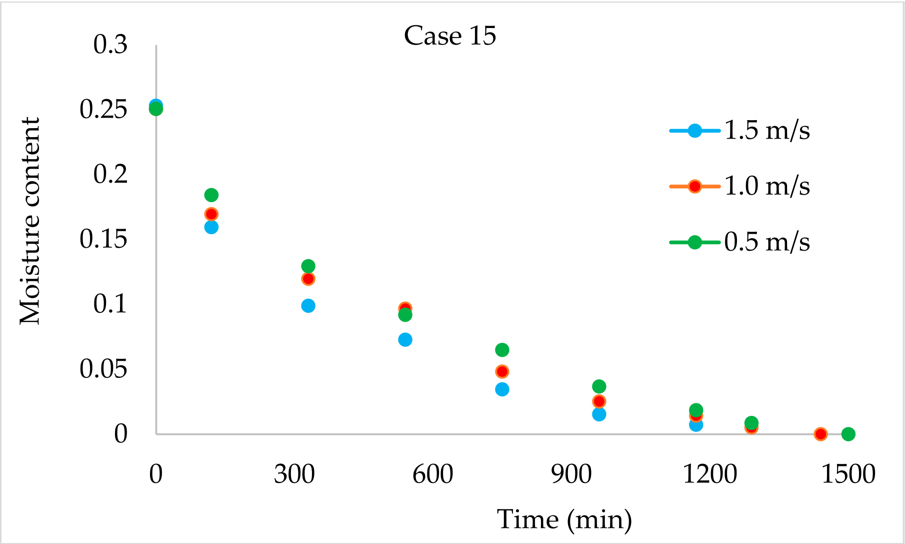

Figure S11. Change of time-dependent moisture content with respect to air velocities during case 15.
